# The human body odor compound androstadienone leads to anger-dependent effects in an emotional Stroop but not dot-probe task using human faces

**DOI:** 10.1371/journal.pone.0175055

**Published:** 2017-04-03

**Authors:** Jonas Hornung, Lydia Kogler, Stephan Wolpert, Jessica Freiherr, Birgit Derntl

**Affiliations:** 1 Department of Psychiatry and Psychotherapy, Medical School, University of Tübingen, Tübingen, Germany; 2 Department of Otolaryngology, Head and Neck Surgery, Medical School, University of Tübingen, Tübingen, Germany; 3 Diagnostic and Interventional Neuroradiology, Universitätsklinikum RWTH Aachen, Aachen, Germany; 4 Fraunhofer Institute for Process Engineering and Packaging IVV, Freising, Germany; 5 Werner Reichardt Centre for Integrative Neuroscience, University of Tübingen, Tübingen, Germany; 6 Lead Graduate School, University of Tübingen, Tübingen, Germany; University of Verona, ITALY

## Abstract

The androgen derivative androstadienone is a substance found in human sweat and thus is a putative human chemosignal. Androstadienone has been studied with respect to effects on mood states, attractiveness ratings, physiological and neural activation. With the current experiment, we aimed to explore in which way androstadienone affects attention to social cues (human faces). Moreover, we wanted to test whether effects depend on specific emotions, the participants' sex and individual sensitivity to smell androstadienone. To do so, we investigated 56 healthy individuals (thereof 29 females taking oral contraceptives) with two attention tasks on two consecutive days (once under androstadienone, once under placebo exposure in pseudorandomized order). With an emotional dot-probe task we measured visuo-spatial cueing while an emotional Stroop task allowed us to investigate interference control. Our results suggest that androstadienone acts in a sex, task and emotion-specific manner as a reduction in interference processes in the emotional Stroop task was only apparent for angry faces in men under androstadienone exposure. More specifically, men showed a smaller difference in reaction times for congruent compared to incongruent trials. At the same time also women were slightly affected by smelling androstadienone as they classified angry faces more often correctly under androstadienone. For the emotional dot-probe task no modulation by androstadienone was observed. Furthermore, in both attention paradigms individual sensitivity to androstadienone was neither correlated with reaction times nor error rates in men and women. To conclude, exposure to androstadienone seems to potentiate the relevance of angry faces in both men and women in connection with interference control, while processes of visuo-spatial cueing remain unaffected.

## Introduction

Human sweat contains chemosignals that have been shown to increase state anxiety in women[1], induce fearful facial expressions in the observer [2] and increase reaction times to anxiety-related words [3]. Though the exact composition of human sweat varies between individuals, it contains a mixture of different compounds including androgens like androstenone, androstanol and androstadienone (4,16-androstadien-3-one, AND) [4]. Especially AND was repeatedly identified in male and female axillary hair [5, 6] and was found to affect mood states as it reduced nervousness and tension [7] and increased positive mood in women [8]. Yet, the effect of AND on mood might be context dependent leading to an increase of negative mood in men and no decline of positive mood in women only in a sad context [9]. Furthermore, sex of the experimenter can possibly have an influence on AND-effects as Lundstrom and Olsson [10] found an increase of positive mood for female participants only when tested by a male but not female experimenter. Interestingly, in the same study sex of experimenter did not have an influence on a task of sustained attention suggesting a dissociation between the effects of sex of experimenter on mood and cognitive parameters.

Apart from its effect on mood, AND has recently been discussed to affect attentional processes as women stated to feel more focused [10, 11] and reported higher pain intensity under AND [12]. In addition, Hummer and McClintock [13] provided initial evidence that under AND exposure a general attentional bias towards emotional stimuli was present in both men and women.

Given its occurrence in human sweat it has furthermore been surmised that AND may especially influence attention in social contexts. Indeed, preliminary research shows that under AND exposure heterosexual women spent more time looking at female faces [14] and rated male faces more attractive [15, 16]. Furthermore both men and women responded more slowly to social negative and quicker to social positive images [17] only under AND exposure. Finally, Frey et al. [18] reported a facilitation of reaction times only to angry faces under AND exposure which was interpreted as an enhanced allocation of attention to threat-related cues.

Given the potential role of AND as a human chemosignal and the fact that human faces are strong social cues it can be expected that AND modulates the salience of human faces in experimental tasks like the emotional Stroop and emotional dot-probe task that tap different attentional processes by using the salience of emotional stimuli [19].

First, the dot-probe task [20] allows to assess attentional biases induced by the presence of emotional and non-emotional stimuli. Specifically, participants see an emotional and non-emotional face to the left or right of a central fixation cross. This pair of faces is then followed by a dot-probe either on the left or right side of the screen. Participants are asked to indicate the position of the dot-probe by a button press assuming that different emotional information presented before shift attention visuo-spatially and thus influence subsequent performance in detecting the dot-probe [21, 22].

In contrast to this, the emotional Stroop task [23, 24] creates a response conflict because of the incompatibility of a target and distractor stimulus. This is similar to the classical Stroop task [25] where participants are asked to name the ink color of a color-word while ignoring the meaning of the word. In the emotional Stroop task two emotionally conflicting stimuli are presented. That is, participants are asked to name the emotion expressed by a face while ignoring the meaning of an overlaying emotional word. Typically, participants react faster and more accurately when the emotional expression of the face and the word match (congruent trial) compared to when they do not match (incongruent trial)., indicating that participants have to resolve a costly emotional conflict [23, 24].

To our knowledge no previous study has used facial stimuli from the same database in both above tasks in the same study. This is necessary to clarify whether a potential chemosignal like AND acts in either a specific or in a general way when seeing social cues like human faces. Furthermore, no previous research has addressed the specificity of AND-action with regard to threatening stimuli. Threat can be signaled both by emotional expressions of anger and fear. However, angry and fearful faces are not identical in the eye of the observer as anger is believed to signal an immediate while fear only points to a potential threat in the environment [26, 27]. For this reason, we incorporated not only happy but also angry and fearful facial expressions disentangle the effects of AND on these emotions.

Based on partial evidence from previous studies [13, 18] we hypothesize that under AND compared to a placebo odor both men and women will allocate more attention to emotional faces, i.e. they will show a stronger attentional bias for emotional compared to non-emotional faces (dot probe task). Second, we expect participants to pay more attention to emotional target faces and less attention to non-relevant emotional words resulting in a weaker emotional conflict in the Stroop task.

Moreover, we expect these effects to be more pronounced for angry compared to happy faces [18]. Furthermore, since sex differences exist with respect to the behavioral relevance of emotional stimuli suggesting higher salience for negative emotions in men [28] and positive emotions in women [29],we further assume stronger effects of AND in men for fearful and angry faces and stronger effects in women for happy faces. Finally, as no former AND study has used two threat related stimuli (anger, fear) in the same experiment our hypothesis is not directional as to whether anger and fear will elicit different behavioral responses in connection with exposure to AND.

Taken together, the goal of this study is to explore how AND affects attention to social cues (human faces). In addition, we wanted to test whether effects depend on specific emotions, the participants' sex and individual sensitivity to smell AND.

## Materials and methods

### Participants

Twenty-eight male and 31 female students of the local university were recruited and measured twice (once under AND, once under placebo-exposure on two consecutive days). Study inclusion criteria were: 18–35 years of age; sexual orientation: heterosexual; native German speaker; not a regular smoker (not more than five cigarettes a week); normosmia as confirmed by an olfactory identification test; not having a history of any psychiatric or neurological disorder or treatment as confirmed via structured clinical interview, SCID (DSM-IV; [30]) and depression inventory, BDI-II [31]. In addition, women were required to have taken oral contraceptives for at least the last six months and not to be in the pill-free week during the experimental days. Intake of oral contraceptives reduces hormonal fluctuations which might otherwise have an influence in tasks of emotional face recognition like former studies have shown [32, 33]. The intake of any other type of hormones or medication were exclusion criteria for both men and women. Subjects were asked not to wear perfume, not to smoke on the days of testing and not to eat and drink anything but water one hour preceding the experiment. One woman and one man had to be excluded because of high self-report depression scores (BDI-II > 18) and another woman was measured during her pill-free week. Thus, 56 participants (29 women] with a mean age of 24.52 years (*SD* = 3.32) were included in the final analysis.

Students from the local University of Tübingen were recruited via flyers and received financial compensation for their participation. They provided written informed consent prior to participation, and the Ethics Committee of the Medical Faculty of the University of Tübingen approved the experimental protocol of the study. The study was conducted between April to June 2016 at the Department of Psychiatry and Psychotherapy, Medical School, University of Tübingen, Tübingen, Germany.

### Odors

Based on similar procedures performed in our group [34, 35], a 250μM solution of AND (purity of AND ≥ 99%; Steraloids Inc., Newport, RI, USA) was prepared, diluted in propylene glycol and masked with 1% musk oil (Sigma–Aldrich, Deisendorf, Germany; please refer to S4 Text for the exact chemical composition of the musk oil used). This concentration close to detection threshold [36] has been shown to increase autonomic arousal in women [37]. In contrast to this, the placebo solution (PLAC) was solely a 1% musk oil solution diluted in propylene glycol. Both solutions were pipetted on a cotton pad with one-direction permeability and attached on the upper lip directly under the nose [1]. Both, the experimenter and the participant were blind to the odor used on the experimental day as each bottle containing AND or PLAC was coded by an independent chemist. Codes were revealed after the end of data collection.

#### Threshold test

To establish individual sensitivity for AND, a three-alternative forced-choice threshold test with seven stairs ranging from a concentration of 0.00001mM to 10mM was conducted [38]. Participants were presented a triplet of odorous flasks, two of which contained an odorless substance (propylene glycole, Sigma-Aldrich) and one contained AND. Participants were asked to indicate the flask that smelled different from the other two. This test was passed if participants gave four consecutive correct answers. If they failed to correctly identify the flask with AND, the next higher concentration was presented until four correct answers were given or the participant failed to detect even the highest concentration (10mM). A higher threshold indicates better individual sensitivity to AND.

#### Discrimination test

Based on similar procedures (e.g. [15, 36]) to assess whether our olfactory mask (1% musk oil) was successful, a discrimination test was designed which consisted of two identical flasks that contained 1% musk oil solved in propylene glycol and a third flask that contained a 250μM solution of AND solved in 1% musk oil and propylene glycol. Participants were asked to name the flask that was different from the other two. This procedure was performed four times in total. Here, higher scores indicate better individual discrimination ability.

The discrimination and the threshold tests were performed on both experimental days. Furthermore, to assess general olfactory ability, we used the 40-item Monell Extended Sniffin’ Sticks Identification Test (MONEX-40; [39]).

### Procedure

**Fig 1:**
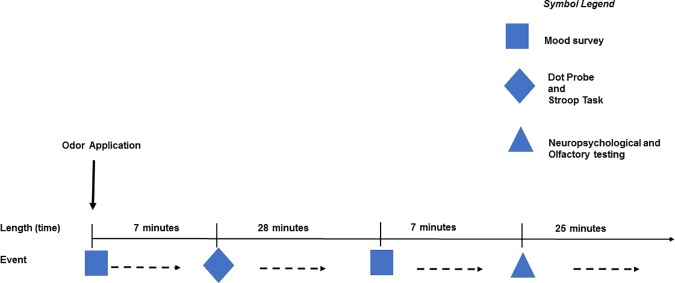
Experimental procedure. After odor application, a mood survey was completed for seven minutes, directly followed by the attentional paradigms lasting for 28 minutes. Another mood survey and olfactory / neuropsychological tests were performed at the end of both experimental days.

Each participant was measured twice on two consecutive days at the same time of day. After filling in the consent form, a structured clinical interview, SCID (DSM-IV; [30]), was conducted to screen participants for mental disorders. Directly following the interview, the respective odor (PLAC or AND in pseudorandomized order) was administered and a mood survey assessed positive affect, negative affect, individual mood items and state anxiety which took about seven minutes. After this, participants completed the dot-probe and Stroop task (order of tasks was randomized across participants]. Then, participants rated each odor with respect to pleasantness, intensity and familiarity on a visual analog scale ranging from 0 (= not at all) to 100 (= very much) and completed a second mood measurement (results of the mood survey can be found in the supporting information S1 Text). The experiment ended with neuropsychological and olfactory assessments lasting for about 25 minutes (see Fig 1 for the procedure). The whole experiment was run by the same male experimenter.

### Neuropsychological tests and self-report questionnaires

To assess basic cognitive abilities, neuropsychological tests tapping verbal intelligence (Wortschatztest, WST; [40]), visuomotor speed and cognitive flexibility via the Trail-making-test (TMT; [41]) as well as selective attention via the Frankfurt Attention Inventory-2 (FAIR-2; [42]) were applied.

Further self-report questionnaires assessed depressive symptoms via the Beck Depression Inventory II (BDI-II; [31]), and alexithymia via the Toronto-Alexithymia Scale-20 (TAS-20; [43]). Female participants also provided information about length of pill intake, brand and any problems due to intake.

### Attention tasks

The order of the emotional dot-probe and emotional Stroop task was counterbalanced across participants. Both paradigms were presented using the software Presentation 17.0 (Neurobehavioral Systems, Albany CA, USA) on an Intel Celeron G1840 desktop computer. Participants were seated approximately 70cm in front of a 21.5inch monitor (BenQ G2222 HDL) with a refresh rate of 60Hz.

#### Emotional dot-probe task (eDOT)

We used a modified version of the original dot-probe task [20] similar to other studies [44, 45]. Emotional facial expressions of 14 different actors (seven women) from all age categories of the FACES database [46] were selected as stimuli. From each of these actors a neutral, angry, happy, and fearful facial expression was selected. Each trial started with the presentation of a white fixation cross on a black background for 500 ms. Immediately afterwards two faces appeared, one on the left and the other on the right side of the screen for 500 ms. Both pictures were replaced by a white dot-probe (0.5x0.5cm), which appeared either on the left or the right side of the display. Participants were asked to indicate the position of the dot-probe as quickly and accurately as possible by pressing the cursor buttons on a standard keyboard. The dot-probe disappeared 1500 ms after onset. The trial ended with the presentation of another fixation cross with a random duration of 750–1000 ms. In each trial, one of the following pairs of faces was presented: neutral-neutral, angry-neutral, fearful-neutral or happy-neutral. Each pair was presented 28 times leading to a total of 196 trials. The location of the emotional face and the location of the subsequent dot-probe were pseudorandomized to ensure that each condition was equally often followed by any other condition. An additional eight trials (one male, one female actor different to the ones used for the task) that showed neutral-neutral faces only were given before the start of the experiment to familiarize participants with the task.

**Fig 2:**
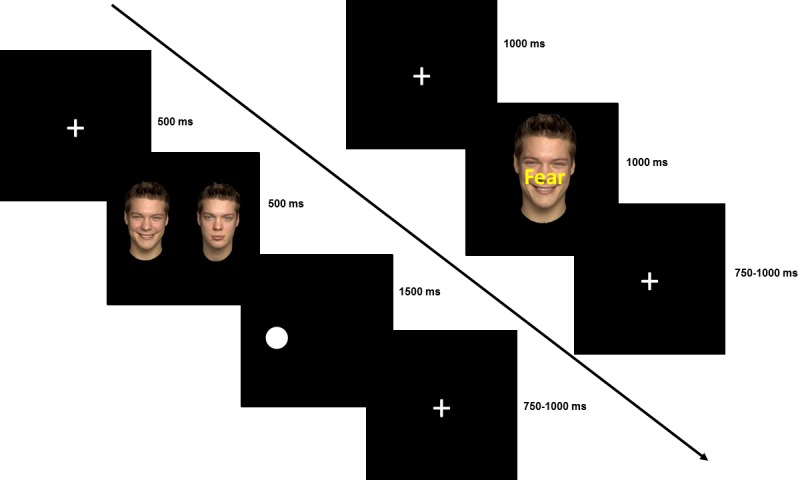
Attention paradigms. *(left)* Procedure of the emotional dot-probe task. The task of the subject was to identify the location of the dot-probe. *(right)* Procedure of the emotional Stroop task. The task of the subject was to identify the emotion of the respective face.

A trial was labeled congruent when the dot-probe appeared at the location of the emotional face and was labeled incongruent when the dot-probe appeared at the location of the neutral face. Thus, for neutral-neutral trials the distinction between congruent and incongruent trials was not made. Please see Fig 2 *(left)* for the visualization of the eDOT.

#### Emotional Stroop task (eSTROOP)

We used a modified version of the emotional Stroop task similar to other studies [23, 24]. Comparable to the eDOT, facial expressions of 12 different actors (six women) from all age categories of the FACES-database [46] were chosen. From each of these actors an angry, fearful and happy facial expression was selected. Each trial started with the presentation of a white fixation cross on a black background for 1000 ms. Then, a face partly covered with an emotional word appeared at the center of the screen for 1000 ms. The presented word could either fit (congruent trial) or not fit (incongruent trial) the facial expression. Participants were asked to indicate the emotion of the face as quickly and accurately as possible via button press. Each trial ended with the presentation of another fixation cross with a random duration of 750–1000 ms.

In each trial one of the following combinations of faces with the emotional words was presented: congruent happy, incongruent happy, congruent fearful, incongruent fearful, congruent angry and incongruent angry. Each of the above combinations was presented 24 times leading to a total of 144 trials (72 congruent, 72 incongruent). The order of trials was pseudorandomized. Twenty-four practice trials (one male, one female actor different to the ones used in the task) including all combinations of facial expressions and emotion words were given before the start of the experiment to familiarize participants with the task. Please see Fig 2 (*right)* for the illustration of the eSTROOP.

### Data preprocessing

For analysis of reaction times (RTs), error trials and trials with reaction times faster than 200 ms (eDOT: *M* = 2.39 trials, *SD* = 3.18 trials; eSTROOP: *M* = 11.89 trials, *SD* = 8.74 trials) were discarded. Afterwards, we defined outliers on a subject to subject basis as deviating two SDs from the mean reaction time of each condition which has been done in a similar fashion for both the dot-probe-task [47, 48] and the Stroop-task [49, 50]. This trimming approach improves the representativeness of mean RTs by excluding trials during which participants were e.g. inattentive or briefly distracted and has been explicitly recommended for the dot-probe-task [51].

Outlier handling led to a further exclusion of trials (eDOT: *M* = 9.11 trials, *SD* = 1.63 trials; eSTROOP: *M* = 6.47 trials, *SD* = 1.28 trials). Thus, in total, 5.9% of the trials in the eDOT and 12.8% of trials in the eSTROOP were excluded. The amount of outliers was comparable between AND and PLAC sessions as shown by a dependent t-test, *t*(54) = 1.22, *p* = .23. Furthermore, the amount of excluded outliers was small compared to a total of 196 stimuli in the eDOT and 144 trials in the eSTROOP rendering only extreme reaction times and only about one trial per condition as outlier.

For analysis of error rates, no trimming could be performed as error rates reflect the sum of incorrect trials divided by the total amount of trials. However, two male subjects were excluded from analysis because of high error rates (> 2 SDs from the male group mean).

### Statistical analysis

Analysis of behavioral data was conducted with the software SPSS 23 (IBM). The statistical threshold was set to *α* = .05. In general, Greenhouse-Geisser corrections were applied if the assumption of sphericity was violated in our mixed-design ANOVAs. Reaction times and bias scores (see below) were logarithmically (log) transformed by help of the formula *y = log10(x+200)*. We decided to log-transform reaction times as values were not normally-distributed (range p-values of the Shapiro-Wilk-test for the eSTROOP: .001-.090; range p-values of the Shapiro-Wilk-test for the eDOT: .002-.189) into normal distribution to perform our parametric mixed-design ANOVA. Log-transformations improved normality considerably (range p-values of the Shapiro-Wilk-test for the eSTROOP: .055-.439; range p-values of the Shapiro-Wilk-test for the eDOT. .040-.915) leaving only one condition in the eDOT at the verge of normality.

As error rates were also heavily non-normally distributed (range p-values of the Shapiro-Wilk-test for the eSTROOP: < .001-.001) we tried several transformations (log-, sqrt-, reverse-score and reciprocal-transformations). However, no transformation was able to confer normality to error rates. We therefore ran our mixed-design ANOVA with untransformed values and computed non parametric post-hoc-tests.

Post-hoc tests and planned contrasts were Bonferroni-corrected for multiple comparisons to a significance threshold of *α* = .05. An effect was labeled trend or marginally significant in case *p*-values between .05 and .09 occurred. Effect sizes were reported in case of significant results for t-tests (Cohen’s *d*) and for F-tests (*η*^2^).

To assess whether men and women were different for any of our neuropsychological tests and self-report questionnaires, independent sample t-tests were conducted between men and women.

### eDOT

Mean reaction times (RT) were first used to calculate the following frequently used attentional bias scores [22, 44]:

*Bias Index (BI) = RT (incongruent trials)–RT* *(congruent trials)*
*Orienting Index (OI) = RT (neutral trials)–RT* *(congruent trials)*
*Disengagement Index (DI) = RT (incongruent trials)–RT* *(neutral trials)*

The BI reflects the general tendency to get distracted by emotional information as positive scores indicate overall longer RTs for incongruent compared to congruent trials. The OI quantifies the tendency to direct attention towards emotional stimuli. Positive scores reflect a stronger orienting to congruent emotional stimuli. Finally, the DI measures the difficulty to withdraw attention once an emotional stimulus has caught attention. Positive scores reflect diminished disengagement from incongruent stimuli. Note that OI and DI will always add up to BI. Each of these attentional bias scores was subject to a 3x2x2 mixed ANOVA including the within-subject factors Emotion (angry, fearful, happy) and Odor (AND, PLAC) and the between-subjects factor Sex (male, female).

#### eSTROOP

Mean RTs and error rates were compared using a mixed 3x2x2x2 ANOVA including the within-subject factors Emotion (angry, fearful, happy), Odor and Congruency (congruent, incongruent) as well as the between-subject factor Sex. As no neutral trials existed in the eSTROOP, we only calculated the emotion-specific Bias Index (RT incongruent trials—RT congruent trials) and performed a 3x2x2 ANOVA with Emotion, Odor and Sex as factors.

#### Olfaction

To investigate whether AND threshold performance and discrimination ability for AND was affected by the experimental day or by participant sex, a 2x2 mixed ANOVA was conducted including the factors Day (AND-day, PLAC-day) and Sex. Furthermore, for discrimination ability separate one-sample t-tests were conducted to check whether men and women differed from chance (i.e. 1.33). Odor ratings (pleasantness, intensity, familiarity) were compared by means of a 2x2 mixed ANOVA including the within-subject factor Odor and the between-subjects factor Sex. To analyze sex differences regarding general olfactory performance (MONEX-40) an independent sample t-test was applied.

#### Mood

For mood states (positive affect, negative affect, state anxiety, individual mood items) 2x2x2 mixed ANOVAs with the factors Odor, Time (baseline, post assessment) and Sex were conducted. Please see supplementary material (S1 Text) for results on mood states.

#### Correlation analyses

To directly compare bias indices between the eDOT and eSTROOP we conducted correlation analyses via Pearson’s correlation coefficient using the log-transformed BI-scores of eDOT and eSTROOP.

To assess whether individual sensitivity for AND and intensity perception of AND was associated with emotion specific RTs and error rates in both tasks, we conducted correlation analyses via Spearman’s Rho. For the eDOT, bivariate correlations were conducted between AND threshold scores / intensity ratings and bias scores separately for all emotions and men and women under exposure to AND and placebo while for the eSTROOP an additional bivariate correlation was conducted between AND threshold scores / intensity ratings and error rates. Spearman’s Rho was selected as AND threshold scores could not be transformed into normal distribution. Please see supplementary material (S2 Text) for results on correlation analyses.

## Results

### Sociodemographic data

Independent sample t-tests confirmed that men and women did not differ regarding age *t*(54) = 1.13, *p* = .26, years of education *t*(54) = 0.96, *p* = .34, verbal intelligence (WST) *t*(54) = 0.56, *p* = .96, trait anxiety (STAI-T) *t*(54) = 0.54, *p* = .96, depression (BDI-II) *t*(54) = 0.88, *p* = .38, alexithymia (TAS-20) *t*(54) = 0.62, *p* = .54, attention (FAIR-2) *p* = .54, *t(*54) = 1.59, *p* = .12, and olfactory identification ability (MONEX-40) *t*(54) = 0.66, *p* = .51. Please see supplementary material (S1 Table) for full results of sociodemographic information.

### eDOT

#### General attentional bias score (BI)

Analyses revealed a significant main effect of Emotion, *F*(1.786, 96.234) = 14.02, *p* < .001, *η*^2^ = .21, indicating that BI scores for fearful faces were significantly larger than for angry (*p* < .001) and happy faces (*p* < .001) while angry and happy faces did not differ (*p* = .62). No main effects of Odor, *F*(1,54) = 0.97, *p* = .33, and Sex, *F*(1,54) = 0.27, *p* = .61, were found. However, a significant Emotion-by-Sex interaction emerged, *F*(1.786, 96.234) = 3.78, *p* = .031, *η*^2^ = .065. All other interactions did not reach significance (*F*s < 2.08, *p*s > .13).

Post-hoc tests disentangling the significant Emotion-by-Sex interaction revealed that men and women did not differ for fearful (*p* = .186), angry (*p* = .570) and happy faces (*p* = 1). Within-sex statistics directly comparing all emotions against each other revealed that in women BI scores were significantly different between fearful and angry (*p* < .001) and fearful and happy faces (*p* < .001) but not between angry and happy faces (*p* = .450). In men, fearful faces differed from happy faces (*p* = .021) but angry did neither differ from fearful (*p* = .420) nor from happy faces (*p* = .930).

#### Attentional orienting (OI)

No significant effects of Emotion, *F*(2,108) = 0.62, *p* = .54, Odor, *F*(1,54) = 0.93, *p* = .34, or Sex, *F*(1,54) = 0.58, *p* = .45, nor any significant interactions of these factors (*F*s < 0.80, *p*s > .37) emerged.

#### Disengagement of attention (DI)

A significant main effect of Emotion was detected, *F*(2,108) = 39.38, *p* < .001, *η*^2^ = .42, showing that DI scores for fearful faces differed significantly from angry (*p* < .001) and happy faces (*p* < .001) but angry did not differ from happy faces (*p* = .780). No main effect of Odor, *F*(1,54) = 0.09, *p* = .77, and Sex, *F*(1,54) = 0.02, *p* = .87, were found. However, a significant Emotion-by-Sex interaction, *F*(2,108) = 3.75, *p* = .027, *η*^2^ = .065, as well as a significant Emotion-by-Odor interaction, *F*(2,108) = 3.57, *p* = .031, *η*^2^ = .062, emerged. No other interaction reached significance (*F*s < 0.28, *p*s > .76).

Post-hoc tests disentangling the significant Emotion-by-Sex interaction indicated that DI scores for fearful faces differed from both other emotions in men and women (*p*s < .001) while those for angry and happy faces did not differ in men (*p* = 1) and women (*p* = .216). Directly comparing DI scores of men and women for each emotion revealed no significant differences (*t*s < 1.36, *p*s > .54).

Post-hoc tests for the significant Emotion-by-Odor interaction directly comparing emotion-specific DI scores for AND versus PLAC did not show significant differences (*t*s < 1.45, *p*s > .14). Comparisons within each odor revealed that for both odors fearful faces differed from angry (*p*s < .009) and happy (*p*s < .001) faces.

#### Overall attentional scores (BI, OI, DI) versus zero

To assess attentional bias indices not only in the framework of our mixed-design ANOVA but to answer the question whether these indices were different from zero, we conducted one-sample t-tests against zero separately for men and women for all attentional bias scores (BI, OI, DI). Only the BI and DI fearful scores in men (BI AND: *p* = .006; DI AND: *p* = .001; DI PLAC: *p* = .004) and women (BI AND: *p* < .001; BI PLAC: *p* = .002; DI AND: *p* < .001; DI PLAC: *p* = .001) were different from zero. Additionally, in men OI happy scores (*p* = .048), DI angry scores (*p* = .035) and by trend OI fearful scores (*p* = .079) under PLAC exposure were different from zero. All other scores did not differ from zero (*p*s > .124) thus attentional biases were most consistently found only for fearful faces.

#### Error rates

As only few errors were made (*M* = 1.2%, *SD* = 1.6%) and error rates under AND exposure did not differ from PLAC, *t*(28) = 0.37, *p* = .71, nor did men differ from women, *t*(54) = 1.3, *p* = .20, no separate ANOVA was conducted for error rates (see Table 1 for the outcome of the eDOT).

**Table 1:** Descriptive statistics of attentional bias scores and RTs for the eDOT (in ms) with SD in brackets.

	MEN		WOMEN	
	AND	PLAC	AND	PLAC
**Bias scores**				
BI happy	1.03 (12.77)	-1.04 (12.81)	1.09 (20.33)	3.73 (16.19)
BI angry	3.78 (16.37)	1.90 (18.09)	-4.29 (20.81)	0.44 (19.96)
**BI fearful †**	13.63 (23.93)*	**2.20 (15.71)**	17.70 (22.04)*	12.08 (18.84)*
OI happy	-1.15 (20.84)	-5.12 (12.83)*	-1.32 (19.06)	0.67 (16.82)
OI angry	3.29 (16.21)	-4.32 (14.13)	-1.05 (31.28)	-0.88 (15.19)
OI fearful	-1.50 (22.96)	-6.46 (18.36)	0.75 (22.18)	-2.24 (18.52)
DI happy	2.18 (19.49)	4.09 (15.71)	2.41 (16.63)	3.06 (19.32)
DI angry	0.50 (18.27)	6.22 (14.54)*	-3.24 (16.12)	1.32 (22.25)
**DI fearful** ‡	15.12 (25.09)*	8.65 (12.25)*	16.95 (18.26)*	**14.32 (20.74**)*
**RTs**				
Happy congruent	412.65 (49.28)	406.85 (59.20)	412.10 (52.55)	416.52 (62.56)
Happy incongruent	413.68 (48.37)	405.81 (56.69)	413.19 (54.60)	420.25 (65.84)
Angry congruent	408.21 (54.86)	406.05 (56.80)	411.84 (60.51)	418.06 (65.56)
Angry incongruent	411.99 (49.65)	407.95 (60.28)	407.55 (49.23)	418.50 (68.93)
Fearful congruent	412.99 (56.51)	408.19 (51.12)	410.04 (52.24)	419.43 (64.31)
Fearful incongruent	426.62 (53.48)	410.38 (57.54)	427.74 (56.18)	431.50 (70.00)
Neutral	411.49 (55.22)	401.73 (56.36)	410.78 (54.32)	417.19 (61.39)

Note. androstadienone (AND); placebo (PLAC)

**†** Men and women showed a greater general attentional bias for fearful compared to angry and happy faces.

**‡** Men and women showed larger disengagement problems for fearful compared to angry and happy faces.

* These scores were significantly different from zero (*p* < .05).

### eSTROOP

#### Reaction times

A main effect of Congruency, *F*(1,51) = 101.02, *p* < .001, *η*^2^ = .67, was detected reflecting faster reactions in congruent compared to incongruent trials. Furthermore, a main effect of Emotion was apparent, *F*(2,102) = 104.21, *p* < .001, *η*^2^ = .67, showing that RTs were faster for happy compared to angry (*p* < .001) and fearful (*p* < .001) facial expressions and faster for angry compared to fearful faces (*p* = .036). No significant effects of Odor, *F(*1,51) = 0.34, *p* = .56), Sex, *F*(1,51) = 0.37, *p* = .55, or any interactions of these factors occurred (*F*s < 2.10, *p*s > .13) (see Table 2 for the outcome of the Stroop task).

**Table 2:** Attentional bias scores, RTs (in ms) and error rates (in %) with SD in brackets for the eSTROOP.

	MEN		WOMEN	
	AND	PLAC	AND	PLAC
**Bias scores**				
Bias happy	41.84 (48.55)	36.94 (40.59)	45.46 (49.81)	42.35 (39.44)
Bias angry	**9.26 (51.63)‡**	44.20 (52.41)	**50.21 (55.93)‡**	51.29 (86.29)
Bias fearful	58.08 (58.60)	47.54 (66.30)	42.61 (76.39)	45.71 (65.41)
**Error rates**				
Error rate happy	3.8 (3.2)	4.8 (4.4)	4.0 (5.4)	3.1 (3.5)
Error rate angry	**10.8 (7.1)†**	8.7 (7.5)	**6.6 (4.0)†**	8.7 (7.3)
Error rate fearful	9.2 (7.7)	8.6 (6.0)	9.6 (8.7)	10.6 (7.7)
**RTs**				
Happy congruent	651.12 (68.62)	657.18 (76.20)	622.10 (71.08)	639.28 (93.26)
Happy incongruent	692.96 (84.78)	694.12 (89.80)	667.56 (92.85)	681.63 (96.32)
Angry congruent	754.22 (74.47)	738.33 (91.07)	731.21(131.02)	737.76(153.92)
Angry incongruent	763.47 (69.83)	782.53 (106.53)	781.43(156.28)	789.05(162.75)
Fearful congruent	754.42 (87.11)	763.97 (112.07)	751.31(140.52	761.98(157.94)
Fearful incongruent	812.50 (96.42)	811.51 (138.68)	793.92(177.19)	807.69(169.12)

Note. androstadienone (AND); placebo (PLAC)

**†** Men made more errors than women for angry faces under AND (significance is based on untransformed error rates).

**‡** Men compared to women showed reduced interference (reaction times) for angry faces under AND (significance is based on log-transformed bias scores).

#### General attentional bias scores (BI)

No main effects of Emotion, *F*(2,102) = 2.08, *p* = .13, Odor, *F*(1,51) = 0.36, *p* = .55, or Sex, *F*(1,51) = 0.30, *p =* .59, were detected. However, a three-way interaction of Emotion–by-Odor-by-Sex, *F*(2,102) = 3.11, *p* = .049, *η*^2^ = .057, occurred. No further significant interactions (*F*s < 2.08, *p*s > .13) emerged.

**Fig 3:**
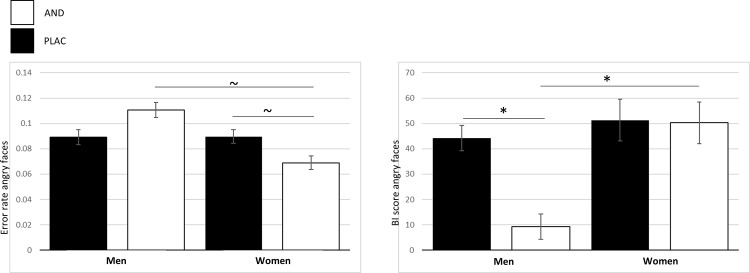
AND- and sex-specific effects for angry faces. (left) Error rates (possible range 0–1) for angry faces are by trend increased for men compared to women under AND and are by trend reduced within women under AND. (right) Bias indices for angry faces depicting reduced interference under AND exposure in men compared to women and reduced interference within men under AND. Significant results are indicated (~ p < .10; * p < .05) and are based on log-transformed values for reaction times (bias index) and untransformed values for error rates. Errors bars indicate two standard errors of the mean (SEM).

To disentangle the significant three-way interaction, emotion-specific 2x2 ANOVAs were conducted. For happy and fearful faces, no significant main effects or interaction were detected (*F*s < 1.00, *p*s > .33). Only for angry faces an Odor-by-Sex interaction was trendwise significant, *F*(1,51) = 3.94, *p* = .053, *η*^2^ = .072, indicating a lower BI score in men compared to women under AND exposure, *t*(51) = 2.77, *p* = .016, *d* = 0.73, but not under PLAC (*p* = 1). Within-sex comparisons showed that men had a lower BI score under AND (*p* = .027) while BI scores for women did not differ between AND and PLAC (*p* = .65) (see Fig 3 *right* for the AND-effect on bias-scores).

#### Error rates

Analysis revealed a main effects of Emotion, *F*(2,102) = 23.66, *p* < .001, *η*^2^ = .32, indicating that fewer errors were made for happy compared to angry (*p* < .001) and fearful faces (*p* < .001), while angry and fearful faces did not differ (*p* = .40). Another main effect of Congruency, F(2,51) = 52.42, p < .001, *η*^2^ = .51, was detected reflecting fewer errors in congruent compared to incongruent trials. Furthermore, a three-way interaction of Emotion-by-Odor-by-Sex emerged, *F*(2,102) = 3.95, *p* = .022, *η*^2^ = .072.

To disentangle the significant three-way interaction emotion-specific 2x2 ANOVAs were conducted. For happy and fearful faces, no significant main effects or interaction were detected (*F*s < 2.33, *p*s > .13). Only for angry faces an Odor-by-Sex interaction was significant, *F*(1,51) = 7.13, *p* = .010, *η*^2^ = .12, indicating that error rates were by-trend higher in men than in women under AND exposure, *U* = 235.50, *z = -2*.*06*, *p* = .080, but not under PLAC (*p* = 1). Within-sex comparisons showed that trendwise women made less errors for angry faces under AND (*p* = .059) while performance in men did not differ between AND and PLAC (*p* = .107 (see Fig 3 *left* for the AND-effect on error rates).

#### Comparing eDOT and eSTROOP via correlating BI scores

Separate correlations for men and women of the BI scores for angry, fearful and happy faces under both odors in the eDOT and eSTROOP yielded no significant correlations, *r*s < .38, *p*s > .354, providing further evidence that a dissociation exists between performance in eDOT and eSTROOP and potential odor effects.

### Olfaction

#### AND threshold

No main effects of Odor, *F*(1,54) = 0.16; *p* = .69, and Sex, *F*(1,54) = 0.02; *p* = .89, nor a significant interaction of Sex-by-Odor, *F*(1,54) = 0.09, *p* = .77, emerged, indicating that AND sensitivity was not different for men and women on both experimental days. For this reason the AND threshold performance was averaged across the two experimental sessions for further analyses.

#### Masking of AND

No significant main effects of Odor, *F*(1,54) = 1.75, *p* = .19; and Sex, *F*(1,54) = 0.32; *p* = .58, or an interaction, *F*(1,54) = 0.01; *p* = .96, occurred indicating that the discrimination ability was not different for men and women on both experimental days. For this reason discrimination performance on both experimental days was averaged for further analyses. Further one sample t-tests against chance indicated that women had detection rates above chance, *t*(28) = 2.06, *p* = .049, *d* = 0.38, while men were above chance only on a trend level, *t*(26) = 1.94, *p* = .064, *d* = 0.38.

#### Odor ratings

For intensity ratings, a significant main effect of Sex emerged, *F*(1,54) = 4.93, *p* = .031, *η*^2^ = .084, indicating higher intensity ratings for both odors in women. The factor Odor did not reach significance, *F*(1,54) = 0.74, *p* = .39. Furthermore, a significant interaction of Sex-by-Odor, *F*(1,54) = 5.82, *p* = .019, *η*^2^ = .097, was detected. Post-hoc tests revealed that men rated AND as less intense than PLAC, *t*(26) = 2.55, *p* = .017, *d* = 0.34, and that men rated AND as less intense than women, *t*(54) = 3.01, *p* = .008, *d* = 0.82. All other comparisons were non-significant (*t*s < 1.02, *p*s > .31).

For pleasantness and familiarity ratings no significant main or interaction effects were observed (*F*s < 2.18, *p*s > .14).

**Fig 4:**
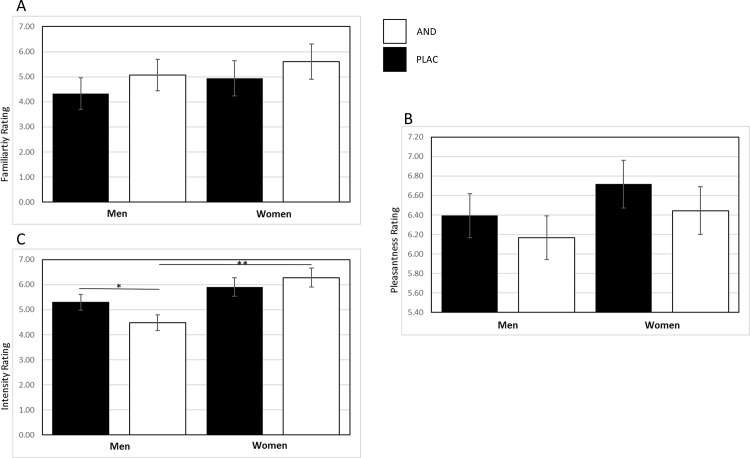
**Odor ratings for Intensity(A), Familiarity (B) and Pleasantness (C).** Men rated AND is less intense than PLAC (* p < .05) and rated AND as less intense than women (** p < .01).

Please see supplementary material (S1 Table) for full results of olfactory assessments and see Fig 4 for all odor ratings.

## Discussion

In this study we aimed at the question whether the putative human chemosignal androstadienone (AND) affects processes of visuo-spatial attention and interference control in connection with social cues (human faces) and whether this effect is modulated by specific emotions, participants' sex and individual sensitivity for AND.

### AND affects interference and error rates in the eSTROOP

In our eSTROOP task we expected participants to pay more attention to emotional target faces and less attention to non-relevant emotional words resulting in a weaker emotional conflict. This expectation was only partially met by our results as sex differences were detected under AND exposure for angry faces. First, only under AND exposure when seeing angry faces, men showed reduced interference, i.e. a smaller difference in reaction times between congruent and incongruent trials.

However, men did not differ with respect to error rates for angry faces between AND and PLAC exposure while this was trendwise the case for women. All in all this may suggest a dissociation between AND-effects on error rates and reaction times. Thus, both men and women seem to be affected with respect to angry faces by AND exposure leading to quicker reactions but unchanged accuracy in men and to an increased accuracy without fastened reactions in women. Former studies point to sex differences regarding the salience of positive and negative emotional stimuli with men reporting anger more often [52], showing stronger emotional experience with angry stimuli than women [53] and displaying larger facilitatory effects to negative emotional primes than women [28]. However, independent of sex, anger stimuli are believed to signal immediate threat [26, 27] and thus possess higher behavioral relevance than e.g. happy or fear cues. Furthermore, in connection with exposure to AND, also Frey et al. [18] found facilitation of reaction times only for angry but not for happy cartoon faces in both men and women.

Taken together, our results give initial evidence that the putative chemosignal AND further potentiates the salience of angry face stimuli for both men and women in a task tapping interference control.

### AND does not affect visuo-spatial cueing in the eDOT

Also in our eDOT task we expected AND to induce a stronger attentional bias for emotional compared to non-emotional faces as has been shown before [13]. However, this hypothesis could not be confirmed as we found no sex- or emotion-specific effects under AND exposure.

Previous studies applying the emotional dot-probe task in healthy participants have provided mixed findings. While Tran et al. [54] found no attentional bias for happy, angry and fearful facial expressions in a mixed sample using a subliminal face presentation time of 50 ms, Pfabigan et al. [44] report a general attentional bias only in men for happy faces under face presentation times of 500 ms. Interestingly, Bar-Haim [55] suggest in a meta-analysis that threat-related stimuli in general do only lead to an attentional bias in clinical samples but not in non-anxious healthy controls. Here we support the idea that neither men nor women show an attentional cueing effect comparing happy to angry faces. However, for fearful faces, both a general attentional bias as well as a difficulty to disengage-effect were found in both sexes plus these biases were significantly different from zero. Thus, both men and women had increased reaction times in incongruent fearful compared to congruent fearful trials and took longer to react in incongruent compared to neutral trials. However, we did not find a significant impact of AND on any of the attentional biases measured with this task. Hence, we do not support findings by Hummer and McClintock [13] who observed a generally increased attentional bias under AND in women and men for happy and angry faces.

However, this null-effect must be taken with caution as the cue presentation time of 500 ms we used in our paradigm might not have been optimal to reveal odor effects. Indeed a wide range of literature has now shown that cue presentation times of 500 ms or shorter lead to a stronger attentional bias. In this respect Bar-Haim [55] reports nearly double effect sizes for short presentation times of 500 ms or shorter compared to cue presentation times of 1000 ms or longer. More recent studies also point out that this effect could be due to an increased number of eye movements during longer presentation times [56] which has been shown to reduce attentional biases in the dot-probe-task or even render them non-significant [57]. To give another example, Koster et al. [47] varied presentation times systematically from 28 to 500 ms and reported increased orienting only towards highly threatening images and a cue presentation time of 100 ms. Thus, it might be that subconscious or very short cue presentation times have greater potential to elicit a strong attentional bias and thereby may be more prone to experimental interventions like AND exposure. In this study we decided for the commonly used face presentation time of 500 ms [22, 44, 58, 59] as also in our Stroop task we had opted for a conscious stimulus presentation time of 1000 ms. Taken together we ask to consider our null-results concerning AND-effects on visual cueing only preliminary and urge to include both shorter cue presentation times and eye-tracking in future studies.

### Why does AND elicit task- and emotion-specific effects?

Given that AND seems to exert an effect on angry facial expressions in the eSTROOP why does this effect not extend to the eDOT?

One explanation may be that both tasks require different attentional and cognitive ressources with the eDOT reflecting a paradigm of exogenous cueing that allows rapid and preattentive shifts of attention in response to the task-relevant dot-probe. In contrast, our eSTROOP is a task that requires volitional executive control and interpretation of faces that are presented for 1000 ms. Thus, in the eSTROOP faces have direct behavioral relevance and need to be interpreted unlike in the eDOT where the reaction is not directed to a facial expression but preattentively to a subsequent non-emotional dot-probe [60, 61].

Considering the question why only anger but not fear elicits AND-dependent effects, there is accumulating evicence that only anger but not fear signals immediate threat and leads to faster reactions [62], better memory encoding [26] and differential neural activation in brain regions like the amygdala [63]. In this connection AND seems to further potentiate the salience of angry faces.

Finally, research on human chemosignals has tried to elucidate the psychological and physiological effects of human sweat [1, 2] and tears [64] which were sampled during fear, disgust and sadness evoking contexts. Indeed, sweat sampled during a disgust and fear evoking context led to a mirrored facial response in the receiver, while tears sampled in a sad context from women led to reduced sexual arousal in men [64]. In our case a synthetically produced compound of human sweat was utilized which was not sampled in a specific context and we thus had no a priori hypotheses regarding an emotion-specific effect. Though this is rather speculative, we suggest that the human sweat compound AND signals an ambiguous social situation and prepares both men and women for a potential conflict by highlighting angry facial expressions.

### Limitations

All our female participants were actively taking oral contraceptives, thus generalization of results to naturally cycling females is not possible. Furthermore, the idea to incorporate two attention paradigms limited the number of presentable stimuli. We therefore used only three emotions: angry, fearful and happy facial expressions. Thus, the question remains whether AND may exert an effect on a wider range of different emotions like disgust or sadness. Furthermore, though AND exerted effects on error rates and the BI score for angry faces in men, these effects were small and must be taken with caution in case of error rates as analyses were carried out with a parametric ANOVA although parameters were not normally distributed. Concerning the eDOT we point to the fact that our cue presentation time of 500 ms may not have been optimal to detect attentional biases and encourage using a shorter SOA in future studies. Finally, as we only used AND versus as a placebo odor further studies including different body compounds or mixtures thereof (i.e. in a natural body odor) are needed to highlight specific and more general effects of chemosensory stimulation during cognitive or emotional processes in women and men.

### Conclusion

In this study we compared men and women taking oral contraceptives in a repeated-measurement design (once under AND, once under PLAC exposure) with respect to visuo-spatial cueing and interference control in connection with facial expressions showing happiness, fear and anger. We provide initial evidence that AND acts in a sex-, emotion and task-specific way as AND exposure potentiated the behavioral relevance of angry faces in both men and women only in the emotional Stroop task. Notably, more automatic processes of visuo-spatial cueing remained unaffected by AND application in women and men.

## Supporting information

S1 Table Sociodemographic information. Description and test-statistics for male and female subjects for sociodemographic data, neuropsychological performance and odor ratings.
(DOCX)

S1 Text Results of the mood survey. (DOCX)

S2 Text Correlation analyses of attentional bias scores of eDOT and eSTROOP with olfactory parameters. (DOCX)

S3 Text Results of order effects of odor application on the eDOT and eSTROOP. (DOCX)

S4 Text Ingredients of the musk oil that was used for masking AND. (DOCX)
