# Supplementary material for: The human body odor compound androstadienone leads to anger-dependent effects in an emotional Stroop but not dot-probe task using human faces
Source: PLoS One. 2017 Apr 3;12(4):e0175055. doi: 10.1371/journal.pone.0175055 (PMC5378404; doi:10.1371/journal.pone.0175055)
Supplement: S1 Table — Description and test-statistics for male and female subjects for sociodemographic data, neuropsychological performance and odor ratings. (DOCX) [file pone.0175055.s001.docx]

|  | **MEN** | **WOMEN** | **t** | **p** |
| --- | --- | --- | --- | --- |
|  | **n = 27** | **n = 29** |  |  |
| Age (years) | 25.04 (3.56) | 24.03 (3.08) | 1.13 | .263 |
| Education (years) | 17.41 (2.40) | 16.88 (1.69) | 0.96 | .343 |
| WST (raw score) | 34.07 (1.75) | 34.10 (2.16) | 0.56 | .956 |
| TMT-A (sec) | 24.59 (6.58) | 21.34 (5.64) | 1.99 | .052 |
| TMT-B (sec) | 39.41 (9.70) | 34.93 (7.59) | 1.93 | .059 |
| TAS-20 (total score) | 41.15 (10.17) | 39.66 (7.94) | 0.62 | .541 |
| BDI-II | 3.93 (3.75) | 3.21 (2.19) | 0.88 | .381 |
| STAI-T | 29.74 (7.45) | 29.83 (4.25) | 0.54 | .957 |
| FAIR-2 (L-index) | 421.09 (87.47) | 463 (93.39) | 1.59 | .119 |
| MONEX-40 | 30.48 (2.64) | 30.93 (2.48) | 0.66 | .513 |
| Threshold AND (mean) | 2.31 (1.70) | 2.40 (1.98) | 0.18 | .860 |
| Discrimination (mean) | 1.65 (0.85) | 1.79 (1.20) | 0.50 | .621 |
| Pleasantness AND | 61.7 (14.8) | 64.4 (21.9) | 0.55 | .587 |
| Intensity AND | 44.8 (24.6) | 62.8 (19.6) | 3.05 | **.004 †** |
| Familiarity AND | 50.7 (25.3) | 56.0 (31.1) | 0.70 | .489 |
| Pleasantness PLAC | 64.0 (12.0) | 67.1 (22.9) | 0.65 | .519 |
| Intensity PLAC | 52.9 (22.9) | 59.0 (22.1) | 1.01 | .315 |
| Familiarity PLAC | 43.2 (22.4) | 49.4 (29.2) | 0.89 | .377 |

*Note*. Verbal intelligence (WST); cognitive speed and flexbility (TMT); alexithymia scale (TAS-20); depression scale (BDI-II); trait anxiety (STAI-T); attention (FAIR-2: L-index); olfactory identification (MONEX-40); androstadienone (AND); placebo (PLAC).

**†** Women rated AND as more intense than men
