# Supplementary material for: The human body odor compound androstadienone leads to anger-dependent effects in an emotional Stroop but not dot-probe task using human faces
Source: PLoS One. 2017 Apr 3;12(4):e0175055. doi: 10.1371/journal.pone.0175055 (PMC5378404; doi:10.1371/journal.pone.0175055)
Supplement: S1 Text — (DOCX) [file pone.0175055.s002.docx]

AND-effects on mood

Positive affect

A main effect of Time was detected, *F*(1,54) = 77.50, *p* < .001, $\eta^{2}$ = .59, indicating a drop of positive affect from baseline to post assessment. No main effect of Odor, *F*(1,54) = 0.77, *p* = .38, or Sex, *F*(1,54) = 0.25, *p*  = .62, was found neither were any interactions significant (*F*s < 2.44, *p*s > .12).

Negative affect

Analysis revealed a significant effect of Time, *F*(1,54) = 8.29, *p* = .006, $\eta^{2}$ = .13, indicating that negative mood was reduced in the post assessment compared to baseline. The effect of Odor was significant by trend, *F*(1,54) = 3.71, *p* = .059, $\eta^{2}$ = .064, indicating that under PLAC negative mood was generally higher than under AND. No main effect of Sex, *F*(1,54) = 0.34, *p* = .56, emerged and no interactions reached significance (*F*s < 1.56, *p*s > .22).

State anxiety

No main effects of Time, Odor or Sex were significant (*F*s < 2.84, *p*s > .098). Only a trend for an Odor-by-Sex interaction, *F*(1,54) = 3.56, *p* = .055, $\eta^{2}$ = .066, emerged. No other significant interaction was detected (*F*s < 1.67, *p*s > .20). Post-hoc tests disentangling the trend interaction indicated that women reported trendwise higher state anxiety under PLAC compared to AND (*p* = .075) while no such effect emerged in men (*p* = .55). Women also reported trendwise higher state anxiety than men under PLAC (*p* = .067) but not under AND (*p* = .79).

Individual mood items

For the items *open*, *energetic*, *social*, *focused* and *happy* only a significant effect of Time, *F*s > 7.88, *p*s < .007, emerged, indicating in all cases a decrease from baseline to post assessment. No significant effects of Odor or Sex, *F*s < 2.53, *p*s > .12, were apparent. In addition, for the item *relaxed* the ANOVA revealed a significant interaction of Odor-by-Time, *F*(1,53) = 6.39, *p* = .014, $\eta^{2}$ = .11. Further post-hoc tests showed that only under AND participants became less relaxed from baseline to post assessment, *t*(54) = 2.67, *p* = .010, *d* = .47, while this decrease did not occur for PLAC (*p* = .93)

Also, for the item *sensual* a trend interaction of Odor-by-Time emerged, *F*(1,53) = 3.71, *p* = .060, $\eta^{2}$ = .065. However, post-hoc tests did not yield any significant findings (*t*s < 1.67, *ps* > .10). No further interaction effects were found (*F*s < 2.54, *p*s > .12).
