# Supplementary material for: The human body odor compound androstadienone leads to anger-dependent effects in an emotional Stroop but not dot-probe task using human faces
Source: PLoS One. 2017 Apr 3;12(4):e0175055. doi: 10.1371/journal.pone.0175055 (PMC5378404; doi:10.1371/journal.pone.0175055)
Supplement: S2 Text — (DOCX) [file pone.0175055.s003.docx]

Correlation analyses eDOT

Sex-specific correlations between individual AND-sensitivity and bias scores or error rates under AND exposure yielded no significant results (*rs* < 31, *p*s > .11) Also, correlations of intensity ratings of AND with bias scores and error rates under AND were non-significant in men and women (*r*s < .-27 , *p*s > .15).

Correlation analyses eSTROOP

Correlations of individual sensitivity to AND with bias scores under AND exposure yielded two significant findings in women: First, with better sensitivity orienting to happy faces decreased, *rs* = -0.41, *p =* .028. Second, with better sensitivity to AND disengagement from angry faces decreased, *rs* = 0.39, *p* = .037. However, these results are only significant by trend as the significance threshold is reduced to $\alpha$ = .017 due to multiple comparisons. No further correlations were significant in men or women (*rs* < -.28, *p*s > .14).

Correlations of individual intensity ratings for AND with bias scores under AND exposure did not reach significance in men and women (*r*s < .30,*p*s > 13).
