# Supplementary material for: The human body odor compound androstadienone leads to anger-dependent effects in an emotional Stroop but not dot-probe task using human faces
Source: PLoS One. 2017 Apr 3;12(4):e0175055. doi: 10.1371/journal.pone.0175055 (PMC5378404; doi:10.1371/journal.pone.0175055)
Supplement: S3 Text — (DOCX) [file pone.0175055.s004.docx]

To investigate whether the application of AND on the first or second day of the experiment had an influence on attentional bias indices, the factor AND-day (AND first day, AND second day) was added as a between-subject factor to our mixed-design ANOVA.

Effects of order of application on the eDOT (BI, OI, DI score)

Only for the OI and DI score a trend-significant three-way-interaction of AND-day x Sex x Odor was detected, OI: *F*(1,52) = 3.54, *p* = .065, $\eta^{2}$ = .064; DI, *F*(1,52) = 3.40, *p* = .071, $\eta^{2}$ = .061, while all other interactions involving AND-day (*F*s < 2.44, *p*s >.100) and the main effects of AND-day did not become significant (*F*s < 0.83, *p*s > .366).

To disentangle the significant three-way interactions, each order (AND first day, AND second day) was explored via 2x2 ANOVAs.

For the OI score the interaction Odor x Sex was only trend-significant when AND was administered on the first experimental day, *F*(1,26) = 3.85, *p* = .062, but not when applied on the second day, *F*(1,26) = 0.45, *p* = .511. The main effects of Sex and Odor did not become significant in either order (*F*s < 2.51, *p*s > .125).

For the DI score neither the interaction Odor x Sex nor the main effects of Sex or Odor were significant in either order (*F*s < 1.85, *p*s > .185).

In case of the OI score further post-hoc tests directly compared OI scores under AND and PLAC when AND was administerd on the first day. Only a trend-significant result was detected within women, *t*(14) = 1.82, *p*s = .092, while no further within- or between-sex-differences were detected (*t*s < 1.83, *p*s > .156).

Effects of order of application on the eSTROOP (BI score)

For the BI score a two-way-interaction of AND-day x Emotion became trend-significant, *F*(2,98) = 2.78, *p* .067, $\eta^{2}$ = 0.54, while no other interactions involving AND-day (*F*s < 1.14 , *p*s > .29) nor the main effect of AND-day became significant, *F*(1,49) = 0.11, *p* = .916.

To disentangle the two-way-interaction each emotion was compared separately within and between the order of AND application. Only when AND was administered on the second day a significant difference between BI happy and BI angry scores emerged, *t*(25) = 2.58, *p* = .048, pointing to a higher difference of incongruent compared to congruent trials for happy target faces only when AND was administered on the second day. No further significant results were detected (*p*s > .369, *t*s < 1.60).
