# Supplementary material for: The human body odor compound androstadienone leads to anger-dependent effects in an emotional Stroop but not dot-probe task using human faces
Source: PLoS One. 2017 Apr 3;12(4):e0175055. doi: 10.1371/journal.pone.0175055 (PMC5378404; doi:10.1371/journal.pone.0175055)
Supplement: S4 Text — (DOCX) [file pone.0175055.s005.docx]

1,4-Dioxacycloheptadecane-5,17-dione (2.5-5 %), Benzyl alcohol (2.5-5 %); Benzyl benzoate (2.5-5%); 2-Aceto-2,3,8,8-tetramethyloctahydronaphthalene (2.5-5 %); Musk grains oil (1-2.5 %)
